# Case Report: Mutation in AIMP2/P38, the Scaffold for the Multi-Trna Synthetase Complex, and Association With Progressive Neurodevelopmental Disorders

**DOI:** 10.3389/fgene.2022.816987

**Published:** 2022-01-24

**Authors:** Mahta Mazaheri, Mahdie Yavari, Hadi Zare Marzouni, Angela Stufano, Piero Lovreglio, Simona D'Amore, Hamid Reza Jahantigh

**Affiliations:** ^1^ Department of Medical Genetics, School of Medicine, Shahid Sadoughi University of Medical Sciences, Yazd, Iran; ^2^ Mother and Newborn Health Research Center, Shahid Sadoughi University of Medical Sciences, Yazd, Iran; ^3^ Dr. Mazaheri’s Medical Genetics Lab, Yazd, Iran; ^4^ Division of Genetics, Department of Cell and Molecular Biology and Microbiology, Faculty of Science and Biotechnology, University of Isfahan, Isfahan, Iran; ^5^ Qaen School of Nursing and Midwifery, Birjand University of Medical Sciences, Birjand, Iran; ^6^ Department of Veterinary Medicine, University of Bari Aldo Moro, Bari, Italy; ^7^ Interdisciplinary Department of Medicine - Section of Occupational Medicine, University of Bari, Bari, Italy; ^8^ Department of Medicine, University of Cambridge, Cambridge, United Kingdom

**Keywords:** leukodystrophies, WES, AIMP2/P38, neurodevelopmental disorders, multi-tRNA synthetase complex

## Abstract

**Background:** Leukodystrophies constitute a heterogeneous group of inherited disorders primarily affecting the white matter of the central nervous system. Aminoacyl-tRNA synthetases (ARSs) catalyze the attachment of an amino acids to their cognate transfer RNAs (tRNAs). Pathogenic variants in both cytosolic and mitochondrial ARSs have been linked to a broad range of neurological disorders, including hypomyelinating leukodystrophies and pontocerebellar hypoplasias (PCH). Aminoacyl tRNA synthetase-interacting multifunctional protein 2 (AIMP2), one of the three non-catalytic components of multi ARS complex, harbors anti-proliferative activity and functions as a proapoptotic factor thus promoting cell death. We report a case of a 7-month-old infant with a complex clinical presentation, including weight loss, severe anemia, skeletal abnormalities, microcephaly and MR imaging features of leukodystrophy with a novel mutation in AIMP2.

**Methods:** Whole-exome sequencing (WES) was performed on the proband. Parental samples were analyzed by PCR amplification and Sanger sequencing.

**Results:** Whole-exome sequencing revealed a novel variant c.A463T in the homozygous state in exon 3 (NM_001,326,607) of AIMP2 [p.(K155X)] in the proband. Parental carrier status was confirmed by target sequencing.

**Conclusion:** Here, we present an Iranian case with leukodystrophy with a novel AIMP2 mutation. This finding broadens the mutational and phenotypic spectra of AIMP2-related leukodystrophy and offers guidance for proper genetic counselling for pre- and post-natal screenings as well as for disease management.

## Introduction

Leukodystrophies are heritable heterogeneous multisystem conditions that primarily affect the white matter of the central nervous system with or without peripheral nervous system involvement (Van Der Knaap et al., 1999). The main neuropathological sites in leukodystrophies are represented by the myelin sheath and myelin-generating cells; however, in some disorders, damage is suspected to originate at the axonal level (Ashrafi and Tavasoli, 2017). The Global Leukodystrophy Initiative (GLIA) Consortium has distinguished on the basis of magnetic resonance imaging (MRI) characteristics two types of classic leukodystrophy: hypomyelinating leukodystrophies, with mild hyperintensity of cerebral white matter found in the T2W sequence of MRI, and demyelinating leukodystrophies, characterized by hyperintensity in T2W and relevant hypointensity in T1W sequences (Parikh et al., 2015; Ashrafi et al., 2020). Moreover, categorization of leukodystrophies based on cellular pathology and metabolic and molecular approaches were proposed in recent years (Van Der Knaap et al., 1999). Currently, the incidence of heritable white matter disorders in pediatric subjects is estimated to be between 1.2/100,000 and 1/6–7,700 live births (Bonkowsky et al., 2010; Numata et al., 2014). Previous studies on leukodystrophies have observed that this condition is often progressive, with nonspecific manifestations and a similar clinical scenario found in most individuals (Vanderver, 2016). Each type of leukodystrophy affects a different part of the myelin sheath and is associated with several different neurological problems. The most common clinical manifestation is the progressive deterioration or regression of the neurological function, with motor deficit due to myelin destruction as most common neurological sign (Vanderver et al., 2015). Typically, hypotonia is more common in the early stages of the disease, especially in hypomyelinating leukodystrophies, whereas a combination of truncal hypotonia and appendicular spasticity is more frequent in the later stages. Signs of involvement of the corticospinal tract (central hypotonia, spasticity), basal ganglia (various types of movement disorders), peripheral nerves (sensory ataxia, abnormal gait), and cerebellar signs (ataxia, nystagmus) are additional important neurological features found in affected individuals (Van Der Knaap et al., 1999). In addition to the neurologic findings, a variety of extra-neural features can be helpful in orientating toward a specific diagnosis. Endocrine disturbances, ophthalmologic, cutaneous, skeletal radiographic abnormalities, dysmorphic facial features, and gastrointestinal symptoms may be detected in patients with leukodystrophy (Parikh et al., 2015).

Although assessment of cerebral white matter involvement by standard brain magnetic resonance imaging (MRI) is the diagnostic tool of choice for leukodystrophy (Van Der Knaap et al., 1999), genetic testing has also taken on a key role in the diagnostic processes of heritable childhood white matter disorders in recent years. Whole-exome sequencing (WES) and whole-genome sequencing (WGS) have been widely used for a better understanding of cases of unknown etiology in several fields of pediatric neurology, with important advantages in terms of predicting possible complications and/or symptoms that may arise during the clinical course of a disease, to identify unexpected clinical presentations associated with genes whose alterations are already known to be pathogenic, to broaden the clinical presentation of already known disorders, to determine prognosis, and finally to identify new genes whose mutations cause disease. (Srivastava et al., 2014).

Aminoacyl-tRNA synthetases (ARSs) catalyze the attachment of an amino acid to its corresponding tRNA, ensuring the translation of genetic information into functional proteins. Pathogenic variants in both cytosolic and mitochondrial ARSs are associated with a wide range of neurological disorders, including hypomyelinating leukodystrophies and pontocerebellar hypoplasia (PCH) (Ashrafi and Tavasoli, 2017). The multifunctional aminoacyl tRNA synthetase-interacting protein 2 (AIMP2, also known as p38), is one of three noncatalytic components (AIMP1, 2, and 3) that form the mammalian multi-tRNA synthetase complex in combination with nine aminoacyl tRNA synthetases. AIMP2 is also involved in other activities besides the multi-tRNA synthetase complex and may determine cell fate through anti-proliferative and pro-apoptotic activities. Specifically, AIMP2 can promote cell death through several modalities. For example, in response to DNA damage it may exert pro-apoptotic activity by modulating p53 activity (Park et al., 2010). In addition, AIMP2 may induce cell death by mediating apoptotic TNF signaling through ubiquitin-mediated destruction of TRAF2 (Choi et al., 2009). An homozygous nonsense variant (c.105C > A; p. Tyr35Ter) of AIMP2 has recently been associated with severe neurodevelopmental alterations (Shukla et al., 2018) like those resulting from other ARS mutations. We herein update the literature and describe a novel AIMP2 variant from one Iranian infant with progressive neurological disorder characterized by lack of development, microcephaly, and skeletal abnormalities. Whole-exome sequencing (WES) revealed a variant c.A463T in the homozygous state in exon 3 (NM_001,326,607) of AIMP2 [p.(K155X)]. MRI of the brain showed global cerebral atrophy and extensive white matter involvement.

## Materials and Methods

### Patients

The proband was referred to Dr. Mazaheri’s lab, Yazd Medical University, Yazd, Iran to confirm the clinical diagnosis. Before being referred for the WES analysis, the proband was reviewed by a metabolic specialist and referred to the lab for the genetic etiology. After written informed consent was obtained from the proband’s parents, a blood sample was collected to perform a WES test. At the time of referral, clinical details and MRI results were provided by the patient’s family. All genomic DNA was isolated from the peripheral leukocytes using a QIAamp DNA Blood Midi kit (Qiagen, Hilden, Germany) according to the manufacturer’s instructions. The DNA samples were stored at −20°C until use. DNA integrity was evaluated by performing 1% agarose gel electrophoresis. Written informed consent was obtained from the patient’s proband for publication of this case report and any accompanying images.

### Clinical Presentation

**FIGURE 1:**
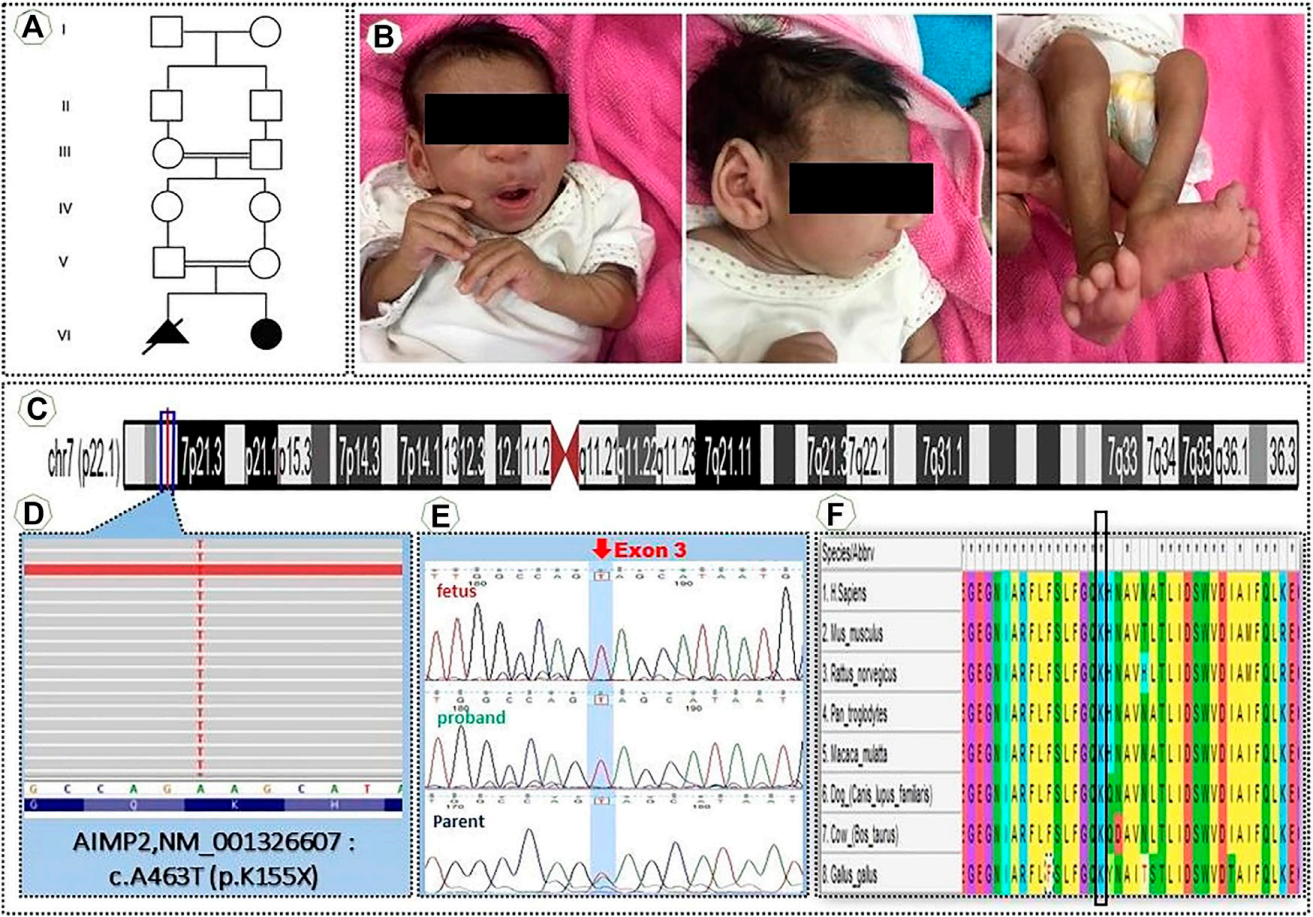
**(A)** Pedigrees of the family **(B)** Clinical manifestations of the proband **(C)** An Integrative Genomic Viewer (IGV) of homozygous nonsense variant AIMP2″ NM001326607 c.A463T, K155X **(D)** Position in the homozygous state in exon 3 of the variant c.A463T (NM_001,326,607) of AIMP2 [p.(K155X)]. **(E)** Sanger sequencing of genomic DNA confirmed the presence of a homozygous mutation in the proband and heterozygous mutation in the parents. **(F)** This alignment shows this position (variant) highly conserved between vertebrates.

A female infant was born by normal vaginal delivery at 34 weeks gestation to consanguineous healthy parents (Figure 1A). Proband’s family did not refers any health problems. The second trimester prenatal ultrasonic screening for fetal malformation didn’t reveal any abnormality. The birth weight was 1,060 g, and her head circumference was 24.5 cm. She was in the neonatal intensive care unit for approximately 25 days after birth due to respiratory failure, hypotension, and clinical features of oligohydramnios. She had delayed cry at birth, microcephaly, and shortened extremities. At 3 months of age, the proband was referred to the medical Centre for a weight reduction of 110 gr. During hospitalization, she received Folic acid tablets, vitamin B6 tablets, pantoprazole tablets and Lanoxin syrup and after 1 week with good health conditions, she was discharged from the hospital. Additionally, karyotype revealed normal female constitution (46, XX). At 7 months of age, because of weight loss, severe anemia, microcephaly, and tilted ankle the proband was referred to our Specialist Centre for further evaluation, including a WES analysis (Figure 1B).

### Radiological Findings

Brain MRI in axial T1WI, T2WI and FLAIR images and sagittal and coronal T2WI were performed. MRI of the brain on a postnatal day 5 showed minor microcephaly, brain atrophy with increased sub arachnoid space, but no changes in the white/gray matter intensity in both hemispheres. A follow-up MRI was performed at 2 months of age and revealed a mildly delayed myelination of brain parenchyma and mild cortical atrophy. At 7 months of age the MRI showed microcephaly, extreme cerebral atrophy, and white matter loss associated with dilatation of lateral ventricle and third vertical. Periventricular white matter hyperintensity in T2/FLAIR in both central hemispheres is seen that could be related to gliosis.

### Whole-Exome Sequencing

Whole-exome sequencing was performed on peripheral leukocyte DNA of the proband. After DNA extraction, exome capture was done using Agilent SureSelect Human All Exon platform following the manufacturer’s protocol (Chen et al., 2015). The quality of FASTQ files was inspected making use of FastQC. Then reads were mapped to GRCh38 utilizing Burrows-Wheeler Aligner (BWA) and duplicates were marked using Picard, complied with by base recalibration, variant calling, and genotyping making use of Genome Analysis Toolkit (GATK). Variations (SNP and INDEL) were filtered based on GATK advised criteria. Ultimately, variants were filtered to maintain those of medical significance. Only variations of exonic or splice site, with less than 1% frequency in the 1,000 Genomes and ExAC databases that were not identified as benign in the clinical data sources passed the filters. These shortlisted annotated variations were further studied for analysis of pathogenic variants. The interpretation of the pathogenicity of the sequence variants is based on the most recent criteria released by the American College of Medical Genetics and Genomics (ACMG) (Richards et al., 2015). Sanger sequencing and PCR using specific primers F-5′-CACCCTTTCCCATGTCATCAG-3′ and R-5′- CCT​TCA​GTT​TAG​CGT​CAT​TCC​A-3 ′ primers for AIMP2 were used to validate the variant in both parents.

## Results

### Genetic Analysis Identified AIMP2 as Candidate Gene

For confirmation of the genetic diagnosis of leukodystrophy, the proband’s DNA was submitted to WES. The number of annotated, potentially pathogenic variants was reduced to 510 complying with bioinformatics filtering, which allows to identify exonic/splicing variants, excluding synonymous and benign sequence variants, and reported homozygous variants with minor allele frequency (MAF) < 0.001. BAM files were converted into variant call format (VCF), and VCF files were used as input for PLINK, a whole genome association analysis toolset, to elucidate the degree of homozygosity from the WES data. This previously unreported mutation most likely influences the AIMP2 [p(K155X)] at exon 3 (Figures 1C,D). This variant was confirmed as homozygous in the proband by bidirectional Sanger sequencing. Sequencing of the parental samples confirmed their carrier status of the novel mutation thus ruling out other causes of apparent homozygosity, such as uniparental isodisomy, allele dropout or copy number variations. (Figure 1E). The Combined Annotation Dependent Depletion (CADD) score, which allows to score the deleteriousness of single nucleotide variants as well as insertion/deletions variants in the human genome, was 42 for this variant (https://cadd.gs.washington.edu/snv), and the allele frequency of this variant in 1,000 genomes of different populaces in heterozygous and homozygous states was 0. (https://gnomad.broadinstitute.org/). This mutation is located in a region that is highly conserved among vertebrates (Figure 1F).

## Discussion

Here we report one case with a phenotype of a severe neurodevelopmental disorder with microcephaly and skeletal radiographic abnormalities with a nonsense variant in AIMP2.

ARSs are essential enzymes that bind specific amino acids to tRNAs prior to protein synthesis. Three non-enzymatic proteins-the ARS-interacting multifunctional proteins (AIMPs)-associate nine different ARSs into a multisynthetic macromolecular complex in higher eukaryotes. Many of these complex-forming ARSs are involved in a wide variety of regulatory processes such as transcription, translation, splicing, inflammation, angiogenesis, and apoptosis. Similar to ARS, AIMPs have functions unrelated to their supporting role in protein synthesis, acting as a cytokine in the control of angiogenesis, immune response, and wound repair, and have crucial regulatory actions in cell proliferation and DNA repair processes (Park et al., 2005). Previous studies have observed associations between several mutations in ARSs and encephalopathies, peripheral neuropathies, and other neurological disorders. In particular, mutations affecting 10 cytosolic ARSs appear to be related to Charcot-Marie-Tooth disease and related neuropathies, whereas mutations affecting 14 mitochondrial ARSs appear to be associated with severe leukoencephalopathies (Ognjenović and Simonović, 2018).

Among auxiliary proteins, p43/AIMP1 has been associated with hypomyelinating leukodystrophy-3 characterized by progressive neurodegeneration, microcephaly, generalized brain atrophy, progressive contractures, and spasticity (Elia et al., 2012; Accogli et al., 2019). AIMP2/p38 is a non-synthetase part of the multi-ARS structure. The p38 protein contains a lysyl tRNA synthetase binding domain, a presumptive leucine-zipper theme, and a C-terminal glutathione S-transferase-like domain, as well as having sequence patterns, which are the binding sites for protein-protein communications (http://www.ebi.ac.uk/InterPro/protein/Q131 55). In addition to its key action in assembling the multi-ARS complex, AIMP2/p38 also is able to suppress cell proliferation by down-regulating c-Myc (Kim et al., 2013). In addition, AIMP2 would also appear to be involved in the pathogenesis of Parkinson’s disease by inducing neural cell death (Ochiai et al., 2021). AIMP2 enhances the ubiquitin-mediated degradation of TNF receptor-associated factor 2, an essential regulator of the tumor necrosis factor-a (TNF-a) signaling pathway, by enhancing the apoptotic response of cells to TNF-a (Choi et al., 2009). In addition, through downregulation of c-MYC it regulates the anti-proliferative activity of transforming growth factor (TGF)-b (Kim et al., 2019). Here we identified a novel pathogenic variant (c.A463T) in AIMP2 [p(K155X)]. To date, only in a single study a nonsense variant in AIMP2 has been described in two unrelated consanguineous families with three affected children each with microcephaly, intellectual disability, seizures refractory to therapy, and spastic quadriparesis (Shukla et al., 2018). MRI showed cerebral, cerebellar, and spinal cord atrophy, with symmetrical T2 hypo-intensities in the bilateral basal ganglia and thinning of the corpus callosum. Whole-exome sequencing of three affected individuals showed c.105C > A (p. Tyr35Ter) variant in AIMP2. In agreement with the previous study, our results suggest that deleterious variants in AIMP2,, might be associated with neurodevelopmental disturbances in humans.

The study has some limitation. First, no functional studies are performed to corroborate the effect of the A463T mutation, such as histochemical staining, RNA extraction and RT-qPCR or Western blot experiments, so it was not possible to evaluate mRNA AIMP2 levels, and possible correlation between mRNA levels and phenotypical presentation. Moreover, the analysis is limited to only one patient.

Despite the limitation, this case confirms the importance of a genetic diagnosis, which provides additional information in the diagnosis of the proband and parents as well as appropriate genetic counselling for the family, including prenatal diagnosis.

## Conclusion

In conclusion, we present a novel AIMP2 mutation in an Iranian infant with clinical and radiological signs of leukodystrophy. The crucial factor in the diagnosis of leukodystrophy is the high importance of medical signs, genetic testing, and MRI findings. Due to the relatively high cost of straight sequencing of genes, these findings could serve for an earlier and definitive diagnosis, which represents a major milestone in the patient’s journey to inform for disease-specific therapies, research eligibility and for symptomatic care.

## Data Availability

The datasets for this article are not publicly available due to concerns regarding participant/patient anonymity. Requests to access the datasets should be directed to the corresponding author.

## Abbreviations

ARSs, Aminoacyl-tRNA synthetases; PCH, pontocerebellar hypoplasias; AIMP2, Aminoacyl tRNA synthetase-interacting multifunctional protein 2; WES, Whole-exome sequencing; GLIA, Global Leukodystrophy Initiative; MRI, magnetic resonance imaging; WGS, whole genome sequencingl; BWA, Burrows-Wheeler Aligner; VCF, variant call format; CADD, Combined Annotation Dependent Depletion; TNF-a, tumor necrosis factor-a.

## References

[B1] Accogli A. Russell L. Sébire G. Rivière J.-B. St-Onge J. Addour-Boudrahem N. (2019). Pathogenic Variants in AIMP1 Cause Pontocerebellar Hypoplasia. Neurogenetics 20 (2), 103–108. 10.1007/s10048-019-00572-7 30924036

[B2] Ashrafi M. R. Tavasoli A. R. (2017). Childhood Leukodystrophies: a Literature Review of Updates on New Definitions, Classification, Diagnostic Approach and Management. Brain Dev. 39 (5), 369–385. 10.1016/j.braindev.2017.01.001 28117190

[B3] Ashrafi M. R. Amanat M. Garshasbi M. Kameli R. Nilipour Y. Heidari M. (2020). An Update on Clinical, Pathological, Diagnostic, and Therapeutic Perspectives of Childhood Leukodystrophies. Expert Rev. Neurother. 20 (1), 65–84. 10.1080/14737175.2020.1699060 31829048

[B4] Bonkowsky J. L. Nelson C. Kingston J. L. Filloux F. M. Mundorff M. B. Srivastava R. (2010). The burden of Inherited Leukodystrophies in Children. Neurology 75 (8), 718–725. 10.1212/wnl.0b013e3181eee46b 20660364 PMC2931652

[B5] Chen R. Im H. Snyder M. (2015). Whole-exome Enrichment with the Agilent Sureselect Human All Exon Platform. Cold Spring Harb. Protoc. 2015 (7), 626–633. 10.1101/pdb.prot083659 25762417 PMC4490097

[B6] Choi J. W. Um J. Y. Kundu J. K. Surh Y.-J. Kim S. (2009). Multidirectional Tumor-Suppressive Activity of AIMP2/p38 and the Enhanced Susceptibility of AIMP2 Heterozygous Mice to Carcinogenesis. Carcinogenesis 30 (9), 1638–1644. 10.1093/carcin/bgp170 19622630

[B7] Elia M. Amato C. Bottitta M. Grillo L. Calabrese G. Esposito M. (2012). An Atypical Patient with Cowden Syndrome and PTEN Gene Mutation Presenting with Cortical Malformation and Focal Epilepsy. Brain Dev. 34 (10), 873–876. 10.1016/j.braindev.2012.03.005 22469695

[B8] Kim J. H. Han J. M. Kim S. (2013). Protein-Protein Interactions and Multi-Component Complexes of Aminoacyl-tRNA Synthetases. Top Curr. Chem. 344, 119–144. 10.1007/128_2013_479 24072587

[B9] Kim M. Kim H. Kim D. Park C. Huh Y. Jung J. (2019). Fluorescence-based Analysis of Noncanonical Functions of Aminoacyl-tRNA Synthetase-Interacting Multifunctional Proteins (AIMPs) in Peripheral Nerves. Materials 12 (7), 1064. 10.3390/ma12071064 PMC6480683 30939730

[B10] Numata Y. Gotoh L. Iwaki A. Kurosawa K. Takanashi J.-i. Deguchi K. (2014). Epidemiological, Clinical, and Genetic Landscapes of Hypomyelinating Leukodystrophies. J. Neurol. 261 (4), 752–758. 10.1007/s00415-014-7263-5 24532200

[B11] Ochiai A. Sawaguchi S. Memezawa S. Seki Y. Morimoto T. Oizumi H. (2021). Knockdown of Golgi Stress-Responsive Caspase-2 Ameliorates HLD17-Associated AIMP2 Mutant-Mediated Inhibition of Oligodendroglial Cell Morphological Differentiation. Neurochem. Res., 1–15. 10.1007/s11064-021-03451-6 34523057

[B12] Ognjenović J. Simonović M. (2018). Human Aminoacyl-tRNA Synthetases in Diseases of the Nervous System. RNA Biol. 15 (4-5), 623–634. 10.1080/15476286.2017.1330245 28534666 PMC6103678

[B13] Parikh S. Bernard G. Leventer R. J. van der Knaap M. S. van Hove J. Pizzino A. (2015). A Clinical Approach to the Diagnosis of Patients with Leukodystrophies and Genetic Leukoencephelopathies. Mol. Genet. Metab. 114 (4), 501–515. 10.1016/j.ymgme.2014.12.434 25655951 PMC4390485

[B14] Park S. G. Ewalt K. L. Kim S. (2005). Functional Expansion of Aminoacyl-tRNA Synthetases and Their Interacting Factors: New Perspectives on Housekeepers. Trends Biochem. Sci. 30 (10), 569–574. 10.1016/j.tibs.2005.08.004 16125937

[B15] Park S. G. Choi E. C. Kim S. (2010). Aminoacyl-tRNA Synthetase-Interacting Multifunctional Proteins (AIMPs): a Triad for Cellular Homeostasis. IUBMB life 62 (4), 296–302. 10.1002/iub.324 20306515

[B16] Richards S. Aziz N. Bale S. Bick D. Das S. Gastier-Foster J. (2015). Standards and Guidelines for the Interpretation of Sequence Variants: a Joint Consensus Recommendation of the American College of Medical Genetics and Genomics and the Association for Molecular Pathology. Genet. Med. 17 (5), 405–424. 10.1038/gim.2015.30 25741868 PMC4544753

[B17] Shukla A. Das Bhowmik A. Hebbar M. Rajagopal K. V. Girisha K. M. Gupta N. (2018). Homozygosity for a Nonsense Variant in AIMP2 Is Associated with a Progressive Neurodevelopmental Disorder with Microcephaly, Seizures, and Spastic Quadriparesis. J. Hum. Genet. 63 (1), 19–25. 10.1038/s10038-017-0363-1 29215095

[B18] Srivastava S. Cohen J. Vernon H. Bara nano K. McClellan R. Jamal L. (2014). Clinical Whole Exome Sequencing in Child Neurology Practice. Ann. Neurol. 76 (4). 473–483. 10.1002/ana.24251 25131622

[B19] Van Der Knaap M. S. Breiter S. N. Naidu S. Hart A. A. M. Valk J. (1999). Defining and Categorizing Leukoencephalopathies of Unknown Origin: MR Imaging Approach. Radiology 213 (1), 121–133. 10.1148/radiology.213.1.r99se01121 10540652

[B20] Vanderver A. Prust M. Tonduti D. Mochel F. Hussey H. M. Helman G. (2015). Case Definition and Classification of Leukodystrophies and Leukoencephalopathies. Mol. Genet. Metab. 114 (4), 494–500. 10.1016/j.ymgme.2015.01.006 25649058 PMC4390457

[B21] Vanderver A. (2016). Genetic Leukoencephalopathies in Adults. Continuum: Lifelong Learn. Neurol. 22 (3), 916–942. 10.1212/con.0000000000000338 PMC5617213 27261689

